# LC–MS/MS application for urine free pyridinoline and free deoxypyridinoline: Urine markers of collagen and bone degradation

**DOI:** 10.1016/j.clinms.2016.08.001

**Published:** 2016-08-21

**Authors:** Jonathan C.Y. Tang, John J. Dutton, Isabelle Piec, Darrell Green, Emily Fisher, Christopher J. Washbourne, William D. Fraser

**Affiliations:** a Bioanalytical Facility, Norwich Medical School, University of East Anglia, Norwich Research Park, Norwich NR4 7UQ, UK; b Department of Diabetes and Endocrinology, Norfolk and Norwich University Hospitals NHS Foundation Trust, Colney Lane, Norwich NR4 7UY, UK; c Department of Laboratory Medicine, Norfolk and Norwich University Hospitals NHS Foundation Trust, Colney Lane, Norwich NR4 7UY, UK

## Abstract

**Background:**

Pyridinium cross-links pyridinoline (PYD) and deoxypyridinoline (DPD) are established markers of collagen degradation. Measurement of PYD and DPD can be used to evaluate changes in bone turnover in patients with metabolic bone disease and to monitor response to anti-resorptive treatment.

**Objective:**

To develop a method to extract and measure urine free PYD (fPYD) and free DPD (fDPD) by liquid chromatography tandem mass spectrometry (LC–MS/MS). The method was used to quantify urine samples from 172 healthy individuals and 63 patients diagnosed with metabolic bone disease.

**Method:**

Acidified urine samples were extracted using solid phase extraction with cellulose slurry. PYD and DPD were separated by reversed-phase, ion-paired chromatography prior to MS/MS detection.

**Results:**

The fully validated method showed good agreement with other laboratories in the UK National External Proficiency Scheme (UK NEQAS). The method was compared against two commercial immunoassays for fDPD and pyridinium cross-links, *r*^2^ were 0.906 and 0.816 respectively. Urine concentrations of fDPD/Cr and fPYD/Cr were significantly higher in the patients than healthy individuals (*p* < 0.001). An average (±SD) fDPD:fPYD ratio of 0.29 (±0.08) was consistently observed across all subgroups. A markedly increased fDPD:fPYD ratio of 8.9 was observed in a patient with type VI Ehlers–Danlos Syndrome (EDS).

**Conclusion:**

Simultaneous measurement of two free pyridinium cross-links provides a valuable, cost effective assessment tool for use in the diagnostic work-up of patients with metabolic bone disease. Improvements in sample extraction efficiency have increased assay specificity and analysis throughput. The use of the fDPD:fPYD ratio can assist in the diagnosis of type VI EDS.

## Highlights

• A novel sample extraction and LC–MS/MS method for parallel analysis of urine fPYD and fDPD.
• Comparable with commercial immunoassays based on validation against industry standard criteria.
• Urine fPYD/Cr and fDPD/Cr significantly higher in patients with high bone turnover disorders.
• Analysis of urine fDPD:fPYD ratio facilitated the diagnosis of type VI Ehlers Danlos Syndrome.

## 1 Introduction

The extracellular matrix in bone consists of 90% type I collagen. The mechanical structure is stabilised by the formation of mature, trivalent cross-links that bond adjacent collagen chains covalently in a triple-helical protein structure. These cross-links are amino acid derivatives of hydroxypyridinium and can be divided into two main groups: pyridinoline (PYD) and deoxypyridinoline (DPD). PYD is present in several connective tissues, whereas DPD is mainly found in bone and mineralised tissue. Bone is constantly remodelled, the coupled process of osteoclastic resorption and osteoblastic formation release many specific biochemical compounds of bone turnover into serum and urine. During the process of bone resorption, the cross-links are released into circulation as free or bound to peptides or sugars, prior to excretion in urine. The amount of pyridinium cross-links in urine reflects the extent of type 1 collagen degradation of the whole body.

Measurement of pyridinium cross-links can be useful in the identification of diseases characterised by increased bone resorption [1]. Assessment of bone turnover status enables the clinician to monitor patient response to anti-resorptive therapy for several metabolic bone diseases [2], [3]. Immunoassays and high performance liquid chromatography (HPLC) methods that quantify free, bound and total (free plus bound) forms of PYD and DPD have been described [4], [5], [6]. Assays are described where total pyridinium cross-links are extracted by acid hydrolysis with concentrated hydrochloric acid added to urine in a pressurised container and heated at temperature between 110 and 180 °C for up to 24 h [7], [8], [9]. Modern chromatographic techniques coupled with fluorescent [10], [11], [12], [13], [14] or UV detection [15] have improved assay sensitivity that allows measurement of the free fraction in urine without acid hydrolysis. An extraction procedure is necessary to remove interfering fluorophores present in the urine matrix before HPLC separation and detection.

Commercial enzyme immunoassays (EIA) are available on automated platforms (Immulite Pyrilinks-D®, Siemens, USA) and 96-well microtitre plate format (Quidel MicroVue, San Diego, CA, USA). The automated Pyrilinks-D® assay for measurement of DPD in urine claims 100% specificity for DPD with 3% cross reactivity with PYD [16], whereas the MicroVue DPD assay claims high affinity for free DPD, with negligible binding to PYD and peptide bound DPD/PYD [17]. The MicroVue pyridinium cross-links assay employs an antibody raised with cross reactivity for both PYD and DPD [18]. In order to obtain final PYD concentration, results must also be assayed for DPD and this subtracted from the total pyridinium cross-links.

Liquid chromatography tandem mass spectrometry (LC–MS/MS) is increasingly becoming the method of choice for quantitative measurement of small molecules. Recent advancements in LC–MS/MS instrumentation have improved the limits of sensitivity and selectivity by orders of magnitude that classical chromatography or immunoassays cannot achieve. Kindt et al. [19] first reported a LC–MS/MS assay for total PYD and DPD in urine and tissue hydrolysates, where samples were acid hydrolysed (6 N HCL overnight at 116 °C) to release peptide-bound PYD/DPD. The debate remains whether the determination of total or free PYD and DPD can provide more information on collagen metabolism in patients. The correlation between free and total collagen cross-links varies by small degrees in chronic joint diseases [5]. In this paper, we describe the use of urinary free DPD (fDPD) and free PYD (fPYD) measurements as markers of bone degradation.

## 2 Materials and methods

### 2.1 Urine sample collection

Method comparisons were carried out on residual urine samples previously collected in the course of routine care. Samples were anonymised to the researchers at point of access, in accordance with generic ethical approval [20]. Random second morning void fasting urines were collected into sterile containers stored at −20 °C and protected from light prior to analysis. Written informed consent was obtained from all study participants, in accordance with the rules of the local ethics committee. Urine samples from healthy individuals (*n* = 172) and patients who presented with metabolic bone disease (*n* = 63) were analysed by the LC/MS–MS method.

### 2.2 Chemicals and reagents

Highly purified PYD/DPD calibration solution and acetylated PYD (INT-PYD) were purchased from Quidel Corporation (San Diego, CA, USA). LCMS grade water and methanol, and analytical grade tetrahydrofuran (THF), heptafluoro-butyric acid (HFBA), butan-1-ol and hydrochloric acid were obtained from Fisher Scientific (Loughborough, UK). Glacial acetic acid was purchased from VWR (Lutterworth, UK). Cellulose microcrystalline was supplied by Alfa Aesar (Heysham, UK).

### 2.3 Calibration standards and quality control material

Human urine pyridinium cross-links calibrator was purchased from Immundiagnostik (Bensheim, Germany). Two urine pools were donated by laboratory staff with permission to be used as internal quality control (IQC) materials. High and low values were obtained and assigned to each pool using the mean value of 20 inter batch replicate analyses. The urine pools were transferred into 1.5 mL microcentrifuge vials and kept frozen in the dark at −20 °C. Aliquots from both pools were assayed in each analytical batch.

### 2.4 Solid phase extraction procedure for fPYD and fDPD in urine

Calibration standard, IQC material or urine samples (0.5 mL) were spiked with 1 mL of freshly prepared acetylated PYD (INT-PYD) in 10 mL glass tubes. Hydrochloric acid (0.5 mL) was added to all tubes, followed by 4 mL of butan-1-ol and 1 mL of cellulose slurry suspended in butan-1-ol/water/glacial acetic acid (4:1:1, v/v) solution. The tubes were capped, mixed by vortex for 30 s followed by 20 end over-end inversion before being left to stand for 30 min at room temperature in a fume hood.

Solid phase extraction procedure was carried out on a PRESSURE+48® positive pressure manifold (Biotage, Uppsala, Sweden) using nitrogen gas supplied from a nitrogen generator (Peak, Scotland, UK) at flow rate of 30 L/min. Acidified urines were transferred into respective empty ISOLUTE® column reservoir (Biotage, Uppsala, Sweden) placed on the positive pressure manifold with 20 μm polyethylene frits pushed to the bottom of each column. Once the urine mixture was allowed to pass through the column, the columns were washed with 6 mL of butanolic solution followed by 0.5 mL THF. During each stage the wash solutions were drained to waste. After each wash step the columns were dried using nitrogen gas pumped at 6 bar of pressure to remove residual wash solutions. Pyridinium cross-links were eluted off into microcentrifuge vials using 0.5 mL of 0.2% HFBA. The eluents collected were centrifuged at 10,000×*g* for 5 min prior to injection into the LC–MS/MS. Using our 48-position positive pressure manifold, a batch of 48 samples can be processed in 2 h.

### 2.5 Liquid chromatography

Urine extracts were injected into a Rheos Allegro UPLC pump (Flux® Instruments, Switzerland) controlled by Janeiro II software. A Waters® 2777 Sample manager (Waters Corp., Milford, MA, USA) equipped with 3-drawer cooler stack regulated at 10 °C was used for sample injection. Chromatographic separation was achieved using the Accucore® (Thermo Scientific, Finland) C18 50 × 2.1 mm, 2.6 μm, reversed-phase solid core particle column heated at 55 °C and protected by a 2 μm, 6.35 mm × 24 mm guard cartridge. A gradient elution profile was set up with column flow rate at 0.4 mL/min. At the start of the gradient the mobile phase consisted of 85%:15% (A) LCMS grade water in 0.1% HFBA and (B) LCMS grade methanol in 0.1% HFBA. The gradient was gradually increased to 99% (B) then returned back to starting gradient in 4.5 min. Solvent divert was employed to divert the ion suppression region of the separation to waste in order to minimise contamination to the source of the mass spectrometer.

### 2.6 Tandem mass spectrometry analysis

LC–MS/MS analysis of PYD, DPD and INT-PYD was performed using Micromass Quattro Ultima Pt mass spectrometer (Waters Corp., Milford, MA, USA) with an integrated electrospray ionisation (ESI) source operated in positive mode. Capillary voltage was kept at 3.0 kV and RF lens 1 and 2 were set at 0.1. Source temperature was maintained at 130 °C. Nitrogen was used as both nebuliser gas at flow rate of 30 L/h and as desolvation gas at flow rate of 850 L/h at 350 °C. Sample cone voltage was 35 kV, collision energies for PYD, DPD and INT-PYD were 40 kV. Argon was used as collision gas. Sample analysis was performed in multiple reaction monitoring (MRM) mode with dwell time of 0.2 s. Transitions of each of the compounds were identified by direct infusion of extracted standards into the ion source via a T-connector. The precursor to product ion transition for PYD, DPD and INT-PYD were 429 > 82, 413 > 84 and 471 > 128, respectively. MassLynx version 4.1 and QuanLynx software (Waters Corp., Milford, MA, USA) were used for system control, data acquisition, baseline integration and peak quantification.

### 2.7 Method validation

Method validation was performed following guidance from the 2013 U.S. Food and Drug Adminstration (FDA) [21] and the 2012 European Medicines Agency (EMA) [22]. The calibration standards were matrix matched to human urine. Quantification of fPYD and fDPD in unknown samples was performed in batches of up to 48 samples, each batch included a set of standards and IQC samples assayed at set points throughout the batch. The concentration of the IQCs ranged from the lowest level (within three times the lower limit of quantification), to the top end of the analytical limit. The IQCs provided the basis of accepting or rejecting the batch, IQC results must be within ±2SD from the mean value. An assay is rejected when IQC failed to fulfil Westgard acceptance criteria [23].

### 2.8 Linearity

Linearity of the method was evaluated by analysing stock standards prepared using Quidel PYD/DPD calibration solutions. PYD concentrations ranged from 0 to 2000 nmol/L and DPD up to 1000 nmol/L were prepared and analysed. The Calibration curve was constructed by plotting the ratio of analyte peak area to INT-PYD peak area against the concentration of their respective standards. Calibration curves were accepted as linear if the weighted linear regression produced a correlation coefficient (*r*^2^) value of >0.990.

### 2.9 Imprecision

Intra and inter-assay imprecision of the method were assessed by running IQC materials twenty times within a single run and separately over a three month period (*n* = 54). Variation was expressed in terms of standard deviation (SD) and coefficient of variation percentage (%CV). The acceptance criteria defined that %CV should not exceed 10% intra-assay and 15% inter-assay across the working range of the assay [21].

### 2.10 Ion suppression

Ion suppression experiment was performed by post column infusion of an 10 μmol/L aqueous mixture of PYD and DPD solution via a T-junction at a flow rate of 20 μL/min, while samples of extracted phosphate buffered saline (PBS) blank and human urine were injected simultaneously via the liquid chromatography system at 0.4 mL/min.

### 2.11 Recovery efficiency

Urine samples (*n* = 10) were aliquoted and spiked with two different concentrations of PYD at 110 nmol/L and 550 nmol/L, and DPD at 45 nmol/L and 250 nmol/L. Assay recovery was determined by calculating the percentage of the measured value against the sum of endogenous value plus spiking concentration.

### 2.12 Lower limit of quantification and detection

Lower limit of quantification (LLOQ) was determined by the lowest concentration quantifiable for which intra-assay precision CV of 20% over 12 replicates and minimum peak signal-to-noise ratio of 10:1 [21]. Lower limit of detection (LLoD) was determined by the lowest concentration that produced a minimum peak area of five times the response produced by the blank [21], [22].

### 2.13 Method comparison

Method comparisons were carried out against two commercially available immunoassays. PYD values obtained from LC–MS/MS were compared against MicroVue pyridinium cross-links enzyme immunoassay (Quidel, San Diego, CA, USA), in 96-well microtitre plate format performed manually using Wellwash automatic plate washer and Multiskan GO spectrophotometer (Thermo Scientific, Finland). DPD comparison was carried out against Pyrilinks-D® solid phase chemiluminescent enzyme-labelled immunoassay analysed on the Immulite® 1000 automated immunoassay system (Siemens, USA).

### 2.14 Urine creatinine measurements

Urine creatinine was measured to obtain fPYD and fDPD to urine creatinine ratio. Samples were analysed using Roche 2nd generation kinetic colorimetric assay based on the Jaffé method performed on the COBAS® C501 analyser (Roche, Burgess Hill, UK). The inter-assay CV ranged from 1.3% to 2.1% and intra-assay ranged between 1.6% and 4% across the assay working range. External quality control (UK NEQAS) return showed an inter-laboratory CV of 2.2%.

fPYD and fDPD results obtained from LC–MS/MS analysis were normalised for variations in renal function and concentrated urine by dividing the cross-links value in nmol/L by creatinine value in mmol/L. The final results were expressed as nmol/L per mmol/L creatinine.

### 2.15 Statistical data analysis

Linear regression analysis and Bland–Altman plots were constructed and analysed by Statistical Package for the Social Science (SPSS) version 22.0.0.1 (IBM, New York, USA). One-way ANOVA was used to compare variables in healthy and patient groups. Significance was defined as *p* < 0.05.

## 3 Results

**Fig. 1:**
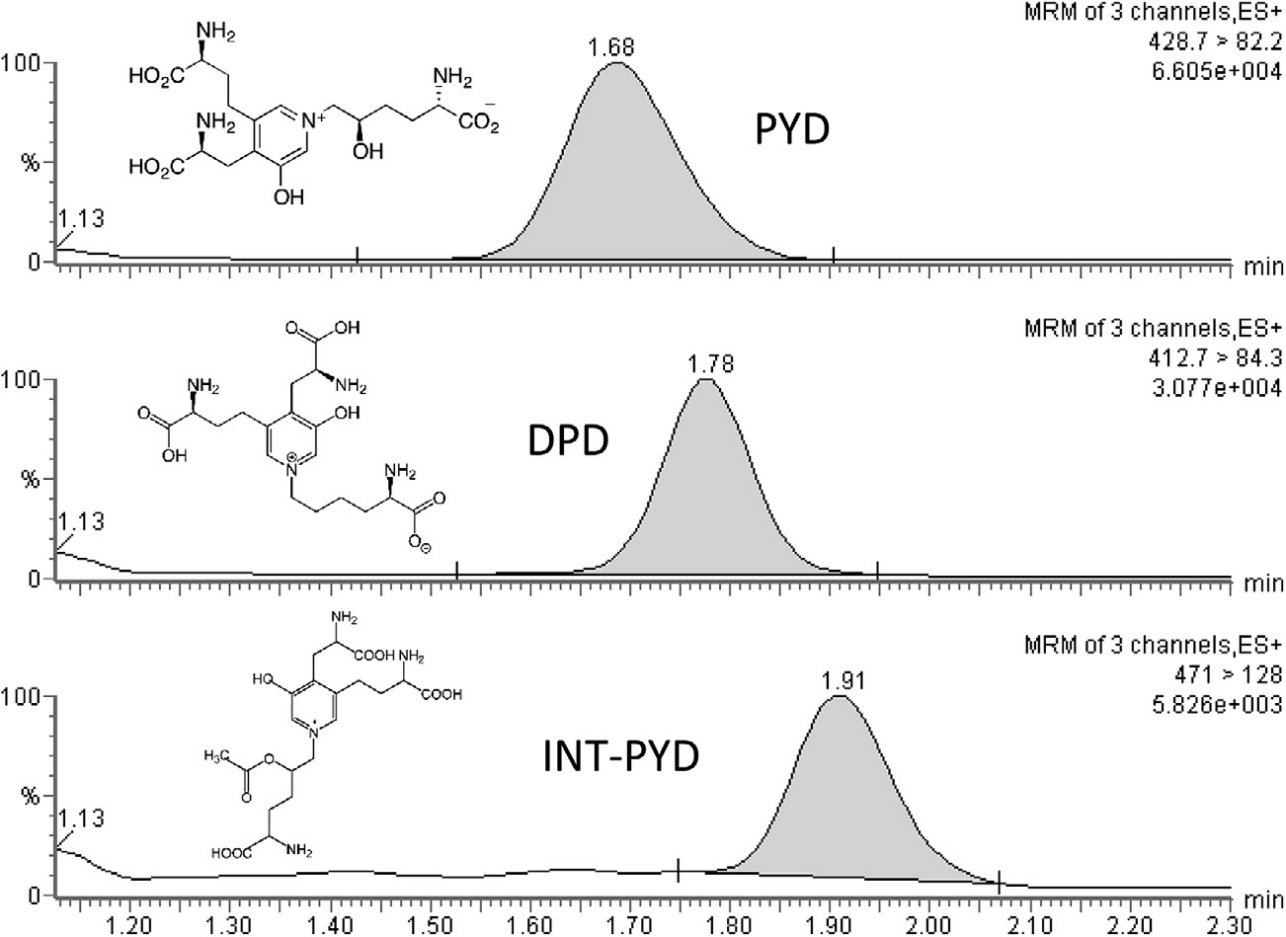
Typical urine sample chromatograms of PYD, DPD and acetylated PYD internal standard following a 30 μL injection. Urine concentrations: fPYD 254.3 nmol/L and fDPD 50.5 nmol/L.

**Fig. 2:**
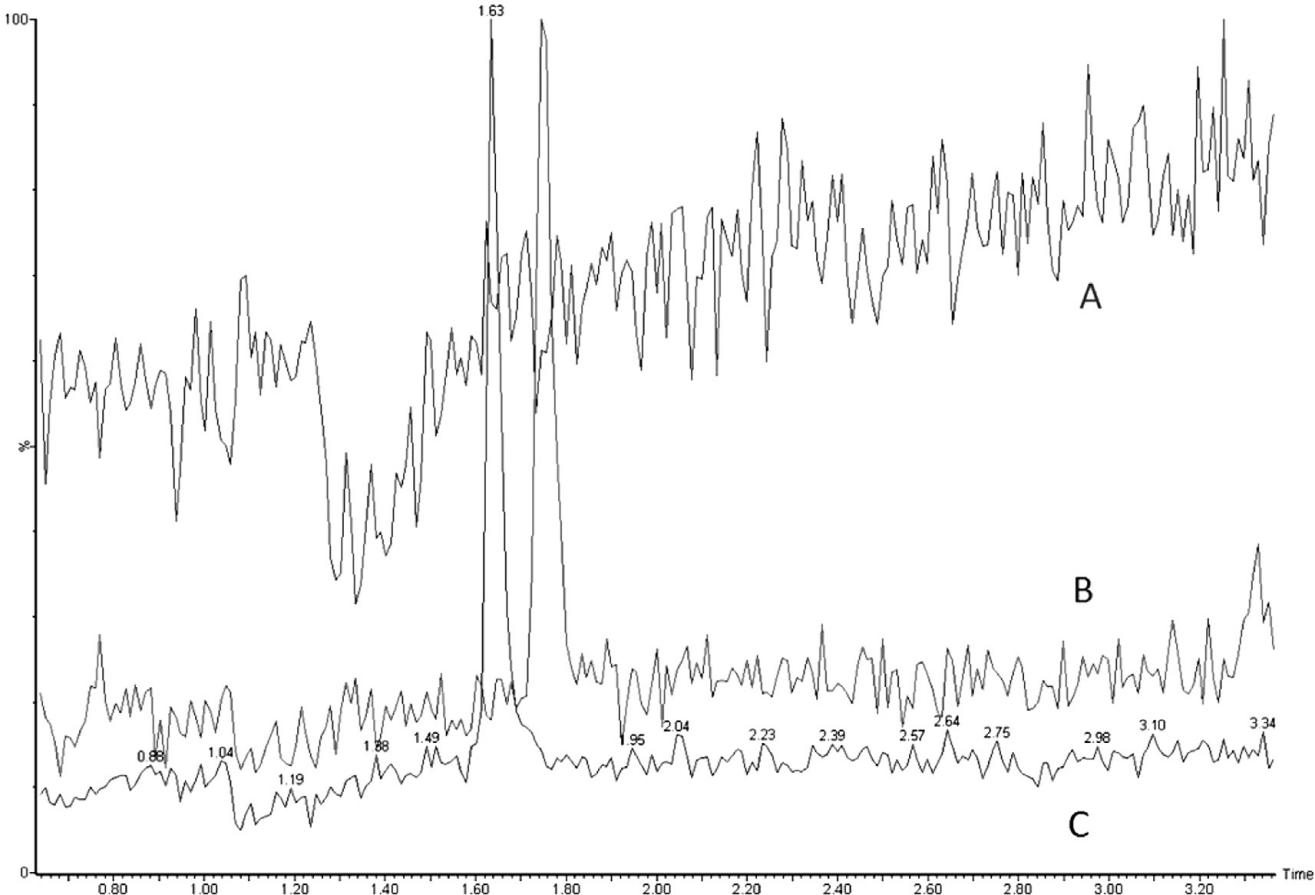
Ion suppression from direct infusion. Reduction in baseline signal was observed in (A) during co-injection of extracted urine sample and post column infusion of PYD and DPD. Ion suppression occurred during 1.1–1.4 min, prior to the elution of (B) PYD, and (C) DPD peaks generated from an extracted urine sample without compound infusion.

PYD, DPD and internal standard peaks were eluted at 1.68, 1.78 and 1.91 min respectively (Fig. 1). The solid core particle column produced clean distinct peaks, minimal baseline noise was observed around the peaks of interest. Ion suppression studies showed the region of interference occurred during the early part of the separation (Fig. 2), solvent divert was subsequently employed to divert the ion suppression region to waste. At the end of each gradient cycle a 100% methanol column wash step was incorporated to remove residual waste. Total run time from injection-to-injection was 4.5 min.

**Fig. 3:**
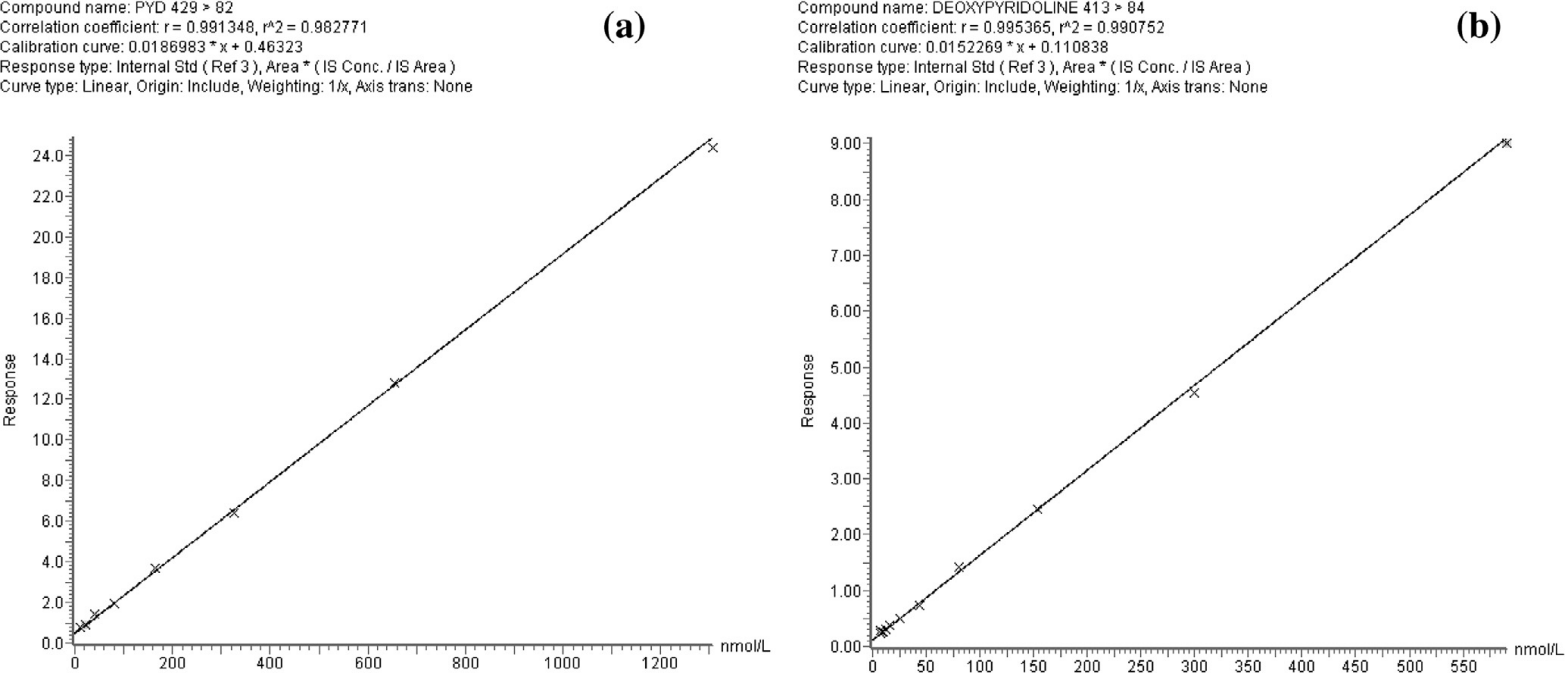
Typical standard curves of (a) PYD, and (b) DPD constructed by plotting the response of each standard on the *y*-axis against their respective concentrations (nmol/L) on the *x*-axis. Analytes response were determined by the ratio of the peak area of the standards to the peak area of the internal standard. Regression analysis showed a typical correlation coefficient *r*^2^ > 0.99.

Standard curves of PYD and DPD (Fig. 3) were generated by plotting the analyte:INT-PYD peak area ratio against the concentration of standards ranging from 0 to 2000 nmol/L for PYD and 0 to 1000 nmol/L for DPD. Linear regression analysis consistently produced *r*^2^ > 0.998. The LLoD and LLoQ for PYD were 5 nmol/L and 6 nmol/L, respectively. DPD were 2 nmol/L and 2.5 nmol/L respectively. The CV of PYD at 6nmol/L was 14.3% and DPD at 2.5 nmol/L was 15.9%.

Imprecision of the method was assessed by analysing two urine pools to establish the intra- and inter-assay variations. Intra-assay imprecision was determined by analysing twenty consecutive measurements of low and high pools. The CVs (mean concentration ±SD) for PYD were 9.1% (75.7 ± 6.9 nmol/L) and 3.8% (739.1 ± 27.9 nmol/L) respectively, DPD were 9.9% (17.9 ± 1.8 nmol/L) and 9.9% (150.7 ± 6.9 nmol/L) respectively. Inter-assay imprecision was determined by analysing the same lot of low and high urine pools over 54 batches during a three-month evaluation period. The CVs (mean concentration, ±SD) for PYD were 10.3% (69.4 ± 7.1 nmol/L) and 10% (747.5 ± 74.7 nmol/L) respectively, DPD were 10% (16.5 ± 1.6 nmol/L) and 13.1% (143.4 ± 18.7 nmol/L).

Extraction recovery was evaluated by determining the amount of cross-links recovered from the amount spiked prior to extraction. With the use of an internal standard, mean recovery for PYD was 106.5% (range 96.4–116.5%) and for DPD was 104.2% (range 102.0–106.3%) (Table 1). The data indicated that the SPE procedure was able to efficiently extract the collagen cross-links from matrix and the method was able to determine the amount recovered accurately. An increase in assay CV to ⩾20% was observed in spiked urine samples extracted without internal standard.

**Table 1:** Recovery efficiency was determined by adding known quantities of (a) PYD and (b) DPD to 10 aliquots of the same urine samples containing endogenous levels of collagen crosslinks. Each aliquot was individually spiked and assayed over separate runs.

Endogenous fPYD (nmol/L)	Spiked (nmol/L)	Mean (±SD) measured value (nmol/L)	Recovered mean (±SD)	% recovery mean (±SD)
1(a)				
151	110	257.1 (±7.3)	106.1 (±7.3)	96.4 (±6.6)
151	550	791.8 (±11.6)	640.8 (±11.6)	116.5 (±2.1)

1(b)				
28	45	73.9 (±4.4)	45.9 (±4.4)	102.0 (±9.9)
28	250	293.7 (±8.9)	265.7 (±8.9)	106.3 (±3.5)

**Fig. 4:**
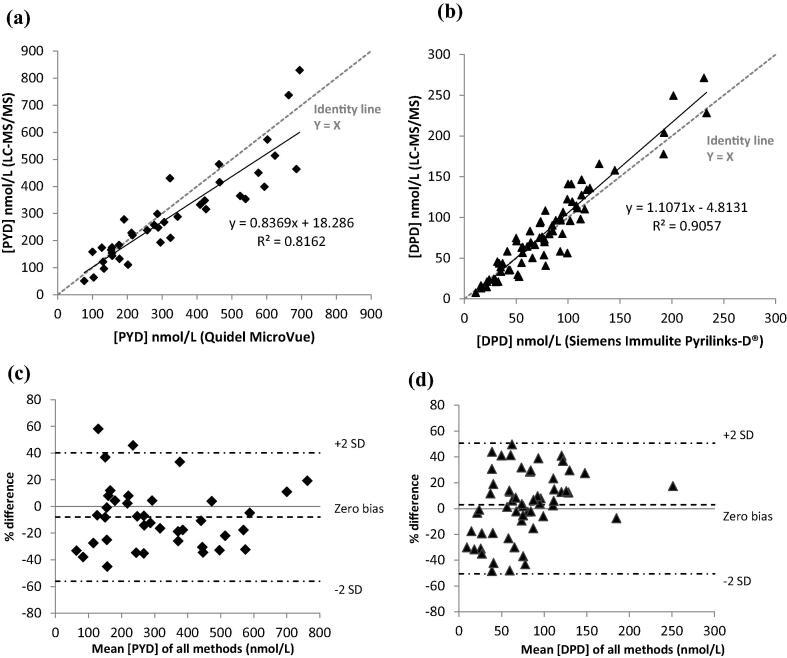
Method of comparison of the LC–MS/MS method with (a) Quidel MicroVue pyridinium cross-links enzyme immunoassay method (*n* = 39), and (b) Siemens Immulite Pyrilinks-D® DPD enzyme immunoassay (*n* = 70). Solid line in the graph represents the fitted regression line; dotted line is the line of identity. Bland–Altman plots comparing the mean concentrations of PYD and DPD against the percentage difference between LC–MS/MS and Immunoassay methods. LC–MS/MS assay showed an average negative bias of −7.94 nmol/L (*r* = 0.903, *p* < 0.0001) for PYD (*n* = 39), and an average positive bias of 3.0 nmol/L (*r* = 0.952, *p* < 0.0001) for DPD (*n* = 70). The dashed lines representing the 95% limits of agreement are shown.

Method comparisons between LC–MS/MS and immunoassays are illustrated by Passing–Bablok regression and Bland–Altman plots (Fig. 4). The line of best fit generated a slope of 0.8369 (*r*^2^ = 0.8162) for PYD, DPD produced a slope of 1.1071 and (*r*^2^ = 0.9057). Bland–Altman plot demonstrated a negative bias of −7.94% (*r* = 0.903, *p* < 0.001) for the PYD values generated by LC–MS/MS method, whereas a small positive bias of 3% (*r* = 0.952, *p* < 0.001) was observed in DPD results between the methods.

**Fig. 5:**
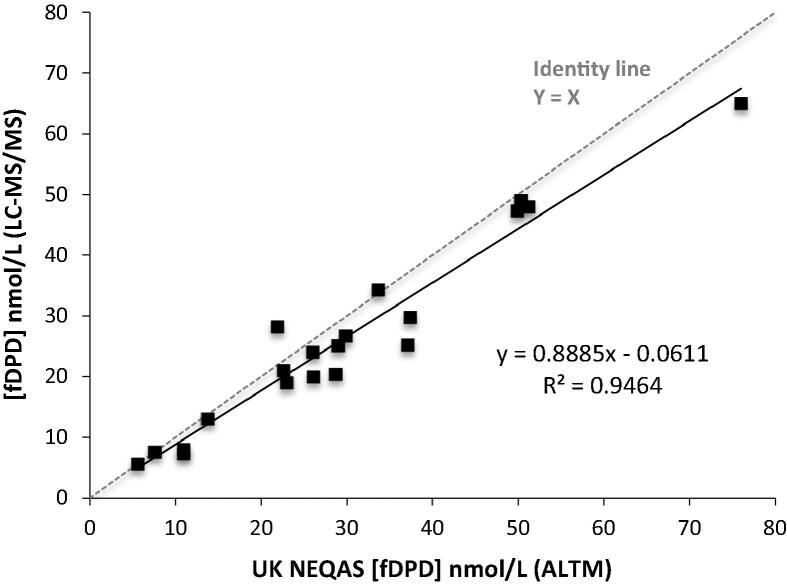
Comparison of urine fDPD returns (*n* = 20) with UK NEQAS all laboratory trimmed mean (ALTM). Solid line in the graph represents the fitted regression line; dotted line is the line of identity.

Materials from external quality controls (UK NEQAS) (*n* = 20) were analysed blind using our LC–MS/MS method. Reports from distributions during 2014–2015 on urine free DPD showed bias ranged between −14.5 and +4.5% compared to all laboratory trimmed mean (ALTM). There were only six participants in the scheme and we were the only LC–MS/MS participant. Our assay showed good agreement (*r*^2^ = 0.9464) with all laboratory trimmed mean (Fig. 5).

**Fig. 6:**
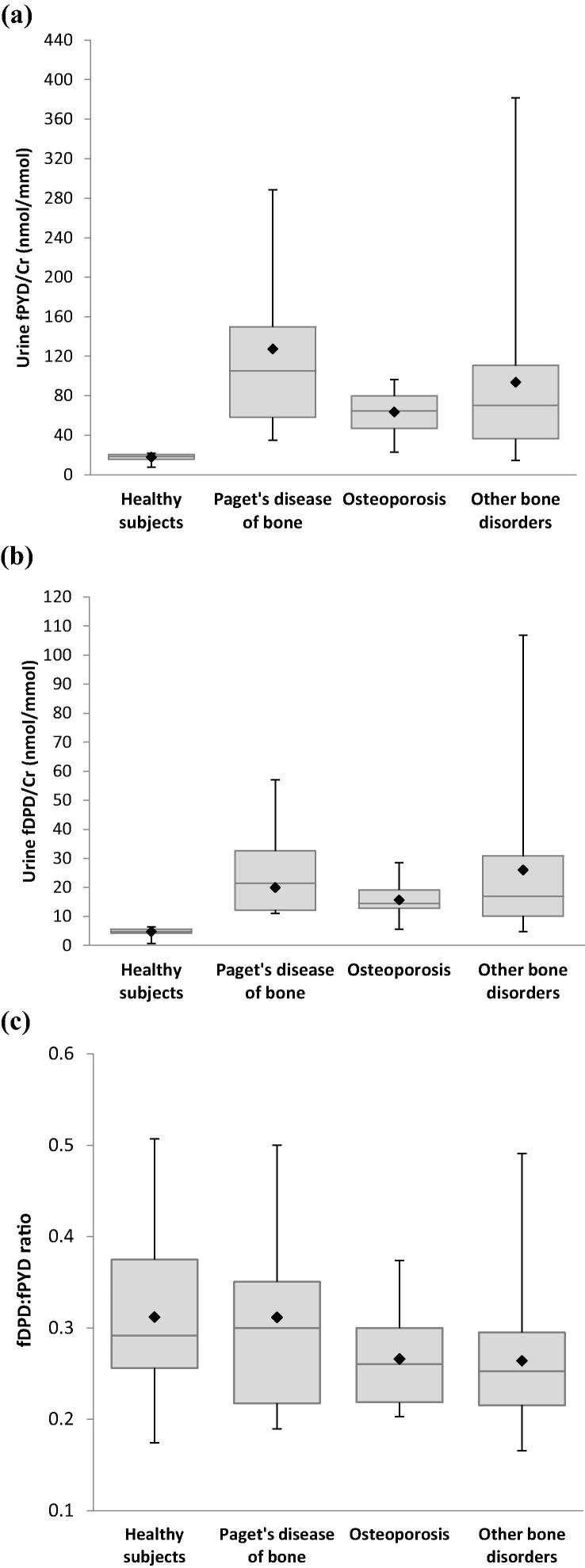
Box and whisker plots showing the concentrations of (a) fPYD, (b) fDPD, and (c) fDPD:fPYD in healthy individuals (*n* = 172) and patients (*n* = 63) diagnosed with Paget’s disease of bone, osteoporosis and other metabolic bone disorders. Urinary concentrations of fPYD and fDPD are expressed as molar ratios with creatinine concentration (nmol/mmol). One way ANOVA analysis showed significant increase in patient groups when compared to healthy subjects. Paget’s disease of bone (fPYD/Cr: *p* < 0.0001, fDPD/Cr: *p* < 0.0001); osteoporosis (fPYD/Cr: *p* < 0.0001, fDPD/Cr: *p* < 0.0001); and other bone disorders (fPYD/Cr: *p* < 0.0001, fDPD/Cr: *p* < 0.0001). fDPD:fPYD ratio showed no significant difference in patient groups when compared to healthy individuals [Paget’s disease of bone (*p* = 0.35), osteoporosis (*p* = 0.28), other bone disorders (*p* = 0.27)]. Results are expressed as mean (♦), median, interquartile range and 95% population interval.

fDPD and fPYD were measured in healthy individuals (*n* = 172) using the LC–MS/MS method, the results were normalised per mmol of urine creatinine. The patient groups (*n* = 63) were divided by the clinical diagnoses into Paget’s disease of bone, osteoporosis, and a subgroup of patients with disorders associated with abnormal bone turnover including osteogenesis imperfecta, osteomalacia, bone fracture and primary hyperparathyroidism. The concentrations of both cross-links were significantly increased in all patient groups (Fig. 6a and b). Despite a substantial increase in cross-links, fDPD:fPYD ratio observed in patient groups did not show a significant difference (*p* > 0.27) from the healthy group (Fig. 6c). During routine healthcare service, we have identified a patient with fDPD/Cr of 722.7 nmol/mmol, fPYD/Cr of 80.8 nmol/mmol with a fDPD:fPYD ratio of 8.9. Review of clinical records showed the patient presented with hypermobility particularly of the joints of the fingers, wrists, ankles, kyphoscoliosis and skin hyperextensibility. The increase in cross-links indicated high bone resorption and collagen degradation. The elevated fDPD:fPYD ratio indicated an abnormal urinary excretion pattern that was consistent with the diagnosis of type VI of Ehlers–Danlos Syndrome (EDS Type VI). Subsequent genetic analysis proved the presence of a homozygous missense mutation of the procollagen-lysine, 2-oxoglutarate 5-dioxygenase 1 (*PLOD1*) gene in exon 13 resulting in decreased activity of lysyl hydroxylase enzyme.

## 4 Discussion

We have developed a robust LC–MS/MS assay for urine fPYD and fDYD. The assay was validated against published acceptance criteria for clinical and research use. In laboratories where there is often a high demand on workload, a multiplex liquid chromatography system can be utilised to perform large batches of samples during the night, allowing technician time to be freed to perform other tasks during the day. LCMS assays using single quadrupole [24] and tandem quadrupole LC–MS/MS [19] instruments have been published. Our method described the use of a triple quadrupole (QQQ) tandem mass spectrometer interfaced with a Z-spray (ESI) source coupled with two radio frequency only ion tunnels. This multi stage process removes unwanted adducts and neutral ions, thus enhances fragmentation efficiency of sample ions and maximises sensitivity. Compound identification based on *m*/*z* detection of precursor to product ion transition is highly specific. The selectivity ensures the assay is not interfered with by other compounds with similar properties of which may co-elute chromatographically. Our method has demonstrated the ability to analyse multiple compounds simultaneously with performance characteristics that satisfy industry standard criteria for method validation. The use of solid core columns has improved peak resolution, signal-to-noise ratio, and increased speed of chromatographic separation. Non porous columns have higher plate count than conventional porous columns due to the high packing density. Our run time of 4.5 min is one of the shortest compared to other documented HPLC methods [4], [11], [13], [24].

Acetylated PYD as an internal standard is commonly used in HPLC methods. Reports of cross talk with PYD possibly due to impure stock material, or due to the removal of an acetyl group during the acid hydrolysis step and/or by MS/MS collision have been documented [19]. We did not encounter such cross-transitional interferences; injections of extracted blank material spiked with acetylated PYD confirmed the absence of PYD and DPD peaks. Inclusion of internal standard is essential to ensure loss of analyte during the analytical process is compensated.

Black et al. [9] first described the use of ion pairing agent to tackle issues with poor column retention due to the hydrophilic nature of PYD and DPD. Heptafluorobutyric acid (HFBA) is a commonly used ion-pairing agent in HPLC assays [4], [11], [13], [24]. However, in LC–MS/MS, the low volatility of HFBA posed a potential problem with source contamination that could result in loss of sensitivity. We kept the amount of HFBA in the mobile phases to a minimum and employed the use of post column divert valve to reduce fouling of the ion source.

Our LC–MS/MS assay was evaluated against two established commercial immunoassays for PYD and DPD and both showed good overall correlation. A negative bias was observed for PYD values generated by LCMS/MS in comparison to MicroVue immunoassay, which is greater at higher concentrations. This finding is expected as the anti-pyridinium cross-links antibody in the MicroVue immunoassay cross reacts with both PYD and DPD [18], whereas the LC–MS/MS method is able to detect one cross-link with zero cross talk. Although LC–MS/MS results correlated with immunoassays in a concentration-dependent manner, the variability of the values observed in the Bland–Altman plots (Fig. 4c and d) is likely to be due to the difference in analyte recovery in urine samples. The use of internal standard in a multiplexed LC–MS/MS method allows for the correction of under/over recovery in each individual sample, such an approach in not possible in singleplex immunoassays.

Using our LC–MS/MS method, we have measured and compared the concentrations of cross-links in a healthy population and in patients with diseases affecting bone turnover. In agreement with previous publications, our findings showed increased levels of fPYD and fDPD in the patient groups [1], [12], [14], [25], [26], [27], [28]. Paget’s disease of bone and osteoporosis are diseases known to have increased osteoclast activity, elevated urine fPYD and fDPD excretion were observed in untreated patients [12]. In patients with vertebral fractures [29], primary hyperparathyroidism [12], cancer related bone disease [30], [31], [32] and thyrotoxicosis [33], [34] there may also be elevated pyridinium cross links up to 2–3 times the upper limit of normal. Baseline measurement of fPYD and fDPD is a valuable tool in the evaluation of bone turnover status in patients and can be a guide to type and severity of disease [12], [35], [36], [37], [38], [39], [40]. A decrease in value post treatment can be observed following bisphosphonate therapy long before a significant change in bone mineral density can be established [32], [34].

The clinical utility of fDPD:fPYD ratio as a diagnostic tool for EDS Type VI is an advantageous aspect of the LC–MS/MS assay. This autosomal recessive disorder can be caused by single nucleotide polymorphism in procollagen-lysine, 2-oxoglutarate 5-dioxygenase 1 (*PLOD1*). Deficiency in the synthesis of resultant enzyme lysyl hydroxylase leads to under-hydroxylation of collagen lysyl residues. Abnormal DPD and PYD formation and excretion pattern results in a marked increase in DPD:PYD ratio as observed in the patient whose sample was referred to our laboratory for analysis. The abnormal ratio is a characteristic feature of patients presenting with EDS Type VI that requires measurement of both cross-links [39], [41], [42]. Simultaneous measurement by LC–MS/MS is more cost effective compared to immunoassays, where samples have to be assayed twice to obtain the required measurements.

In summary, we have developed and validated a method to measure urine fPYD and fDPD using an LC–MS/MS instrument that is widely available in clinical and research laboratories. Based on previously published methodologies, we have made improvements in both sample extraction and detection techniques to increase assay quality and efficiency. The availability of this assay provides a valuable, cost effective diagnostic tool for patients with diseases affecting bone turnover and disorders of collagen metabolism.

## Author contributorship

Development and validation of the method: J.C.Y.T. Conception of the method: J.C.Y.T., J.J.D. and W.D.F. Performed the experiments: J.C.Y.T., I.P., D.G., E.F. and C.J.W. Performed analysis of the data: J.C.Y.T. and I.P. Wrote the manuscript: J.C.Y.T. Revised and approved the final version of the manuscript: all the authors.

## Declaration of conflicting interest

The authors declare no conflict of interest.
